# Disability in cluster headache is more than attack frequency - results from and validation of the English version of the Cluster Headache Impact Questionnaire (CHIQ)

**DOI:** 10.1186/s10194-024-01838-8

**Published:** 2024-08-05

**Authors:** Katharina Kamm, Andreas Straube, Mark Burish, Ruth Ruscheweyh

**Affiliations:** 1 Department of Neurology, University Hospital, Ludwig-Maximilians-Universität München, Marchioninistraße 15, Munich, 81377 Germany; 2 Department of Neurosurgery, UTHealth Houston, Houston, TX USA

**Keywords:** Cluster headache, disability, CHIQ, questionnaire, patient-reported outcome measure

## Abstract

**Background:**

Cluster headache (CH) is associated with high disability. The Cluster Headache Impact Questionnaire (CHIQ) is a short, disease-specific disability questionnaire first developed and validated in German. Here, we validated the English version of this questionnaire.

**Methods:**

The CHIQ was assessed together with nonspecific headache-related disability questionnaires in CH patients from a tertiary headache center and an American self-help group.

**Results:**

155 active episodic and chronic CH patients were included. The CHIQ showed good internal consistency (Cronbach’s α = 0.91) and test-retest reliability (ICC = 0.93, n = 44). Factor analysis identified a single factor. Convergent validity was shown by significant correlations with the Headache Impact Test™ (HIT-6™, *ρ* = 0.72, *p* < 0.001), the Hospital Anxiety and Depression Scale (HADS depression: *ρ* = 0.53, HADS anxiety: *ρ* = 0.61, both *p* < 0.001), the Perceived Stress Scale (PSS-10, *ρ* = 0.61, *p* < 0.001) and with CH attack frequency (*ρ* = 0.29, *p* < 0.001). Chronic CH patients showed the highest CHIQ scores (25.4 ± 7.9, *n* = 76), followed by active episodic CH and episodic CH patients in remission (active eCH: 22.2 ± 8.7, *n* = 79; eCH in remission: 14.1 ± 13.1, *n* = 127; *p* < 0.001). Furthermore, the CHIQ was graded into 5 levels from “no to low impact” to “extreme impact” based on the patients’ perception. Higher CHIQ grading was associated with higher attack and acute medication frequency, HIT-6™, HADS and PSS scores.

**Conclusion:**

The English version of the CHIQ is a reliable, valid, and disease-specific patient-reported outcome measure to assess the impact of headaches on CH patients.

## Introduction

Cluster headache (CH) is a severe primary headache disorder with excruciating unilateral headache attacks lasting 15-180 min and occurring as either episodic CH (with headaches at least every other day for weeks to months followed by remission of >3 months) or, less often, as chronic CH (with either no remission period or remission periods lasting <3 months in the last year) [1]. 

CH is associated with high disability. In the past, this was mostly assessed using migraine-specific disability questionnaires like the Migraine Disability Assessment (MIDAS) [2], general headache disability questionnaires like the Headache Impact Test™ (HIT-6™) [3], or even general (i.e. non-headache) quality of life questionnaires like the SF-12 Health Survey (SF12v2®) [4]. It was criticized that these questionnaires might not capture the real burden of the disorder since CH-specific characteristics are not evaluated and timeframes of weeks to months may not be appropriate for a disorder with a rapid change in attack frequency [5]. 

To address this need, several CH-specific questionnaires have been developed, including the Cluster Headache Quality of Life Scale (CHQ, 28 items), the Cluster Headache Scales (CHS, 36 items, containing a disability subscale) and the Cluster Headache Impact Questionnaire (CHIQ, 8 items) [6–8]. 

Among these instruments, the CHIQ stands out for its brevity (8 items), making it a valuable tool to capture current CH-related disability both for clinical practice and research [7]. Two of the CHIQ items ask for CH-associated limitations in work and family life. Four items assess disability associated with concentration difficulties, irritability, fatigue due to nocturnal attacks and poor predictability of headache attacks. Further, CH-associated self-injurious behavior and the patient’s impression of being a burden to his or her social environment is assessed. Items are rated on a 6-point Likert scale from 0 (“never”) to 5 (“always”), resulting in total scores from 0 to 40. A timeframe of 1 week was chosen to capture current impact and the rapid changes that can occur when patients enter a remission period. To complete the picture, two additional questions assess attack frequency and acute medication use within the last week.

The CHIQ was first developed in German. The validation study of the German version showed good internal consistency and test-retest reliability as well as significant correlations with the HIT-6™, with attack frequency, and with depression, anxiety and stress. CHIQ scores were significantly higher in patients with active CH compared to patients with CH in remission, and significantly higher in patients with chronic CH compared to patients with active episodic CH [7]. In the meantime, an Italian version has been validated with similar results [9]. The aim of the present study was to validate the English language version of the CHIQ.

## Methods

### Study procedure

The CHIQ was translated to the English language using a standard forward-backward translation procedure and was published together with the original German version [7].

The present study was approved by the Institutional Review Board at UTHealth Houston and conducted in accordance with the Declaration of Helsinki. Participants were recruited between September 2022 and September 2023 through one of two methods: the clinic of one of the authors (author M.J.B.) in Houston, Texas, USA, and a CH community support group (Clusterbusters). Inclusion criteria were participants aged ≥ 18 years old with either an ICHD-3 diagnosis of episodic or chronic CH from direct interview with a headache specialist (author M.J.B.) or an ICHD-3 diagnosis of episodic or chronic CH based on review of the clinical characteristics and the ICHD-3 criteria indicated in the headache questionnaire. Exclusion criteria were participants with incomplete data, specifically incomplete clinical characteristics such that the diagnosis could not be confirmed, or incomplete data for the CHIQ. If participants failed to complete one or more of the three validated scales of disability, depression and stress (discussed further below), they were excluded only from the respective analysis.

After informed consent, participants were asked to participate in a baseline online survey, followed by two follow-up surveys after 2 weeks and 3 months, respectively. For the present analysis, the baseline and the 2-week results were used. The questionnaire was administered online via RedCap (Research Electronic Data Capture) [10, 11]. The baseline survey comprised the CHIQ, a thorough headache questionnaire assessing the ICHD-3 criteria for CH and CH treatment, and assessment of comorbidities. Furthermore, the survey included validated questionnaires on headache-related disability (HIT-6™), depression (Hospital Anxiety and Depression Scale (HADS)), and stress (Perceived Stress Scale (PSS-10)) [4, 12–14]. Finally, a single item “How would you rate the impact of cluster headache on your life during periods with headache attacks?” with a rating from 0 to 4 (not at all, a little bit, moderate, quite a bit, extreme) was included to support establishment of a grading of the CHIQ.

The follow-up surveys started with a short questionnaire assessing changes in CH severity or treatment, and comorbidities. Further, the CHIQ, the HIT-6™, HADS and PSS-10 were included. Data from the baseline survey and the first follow-up were used for the present analysis.

### Statistical analysis 

Demographics and CH characteristics are presented as descriptive statistics (mean ± SD or numbers and percentages of patients). The Shapiro-Wilk test was used to evaluate normality of data distribution. Exploratory factor analysis (oblimin principal axes factor analysis, PFA) was performed after confirmation of suitability using the Kaiser-Meyer-Olkin (KMO) criterion and Bartlett test. Item statistics comprising item difficulty and item-scale correlations were assessed. For internal consistency, Cronbach’s alpha was calculated and a value > 0.80 was accepted as good [15, 16]. Test-retest reliability was assessed using intraclass correlation coefficients (ICCs, two-way mixed effect model with absolute agreement for single measures) [17]. Convergent validity between the CHIQ score, CH characteristics and the results of other questionnaires was assessed using Spearman correlations.

Group differences between episodic and chronic CH patients were assessed using a Kruskal-Wallis-ANOVA followed by Bonferroni-Holm correction for three comparisons and to assess differences between CHIQ grades.

Statistical analysis was performed using SPSS Statistics 26 (IBM Corp., Armonk, NY, USA). Significance was accepted at *p* < 0.05 (two-tailed). 

## Results

### Participants

**Fig. 1:**
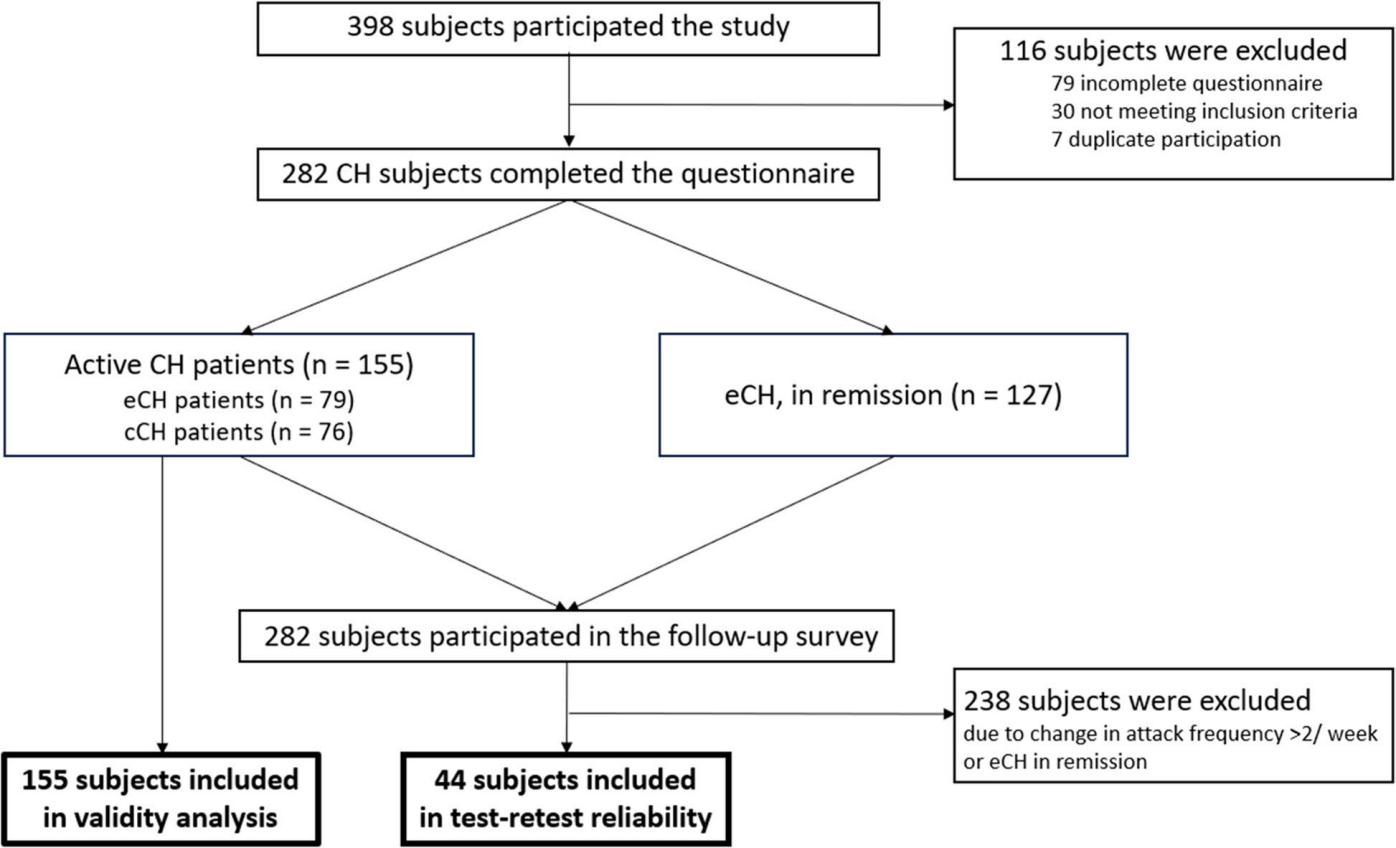
Participant disposition. ‘Active CH patients’ (*n* = 155), meaning active episodic CH and chronic CH patients, were included in the analysis of reliability and validity. After 16.1 ± 3.2 days patients participated a second survey to evaluate test-retest reliability. Participants with active CH at both surveys and a change in attack frequency ≤ 2 attacks per week were included in the analysis of test-retest reliability. Abbreviations: CH, cluster headache; cCH, chronic cluster headache; eCH, episodic cluster headache

398 patients participated in the survey between September 2022 and September 2023. Of these, 116 were excluded due to incomplete data (*n* = 79), non-fulfillment of inclusion criteria (*n* = 30), or duplicate participation (*n* = 7). Of the 282 remaining participants, 206 fulfilled criteria for episodic CH (150 males; age 54.0 ± 13.8 years) and 76 fulfilled criteria for chronic CH (42 males; age 53.9 ± 12.1 years). Patient disposition is shown in Figure 1 and patient characteristics are shown in Table 1.

**Table 1:** Clinical characteristics of included participants. Parentheses indicate percent of n within each of the four columns.  *eCH patients in remission were asked the use and number of preventive medication during the last CH episode. Abbreviations:  CH, cluster headache; cCH, chronic cluster headache; eCH, episodic cluster headache

	**Active CH patients**			**eCH patients, in remission**
	**Total active CH patients**	**cCH patients**	**Active eCH patients**	
**N**	155 (male=106)	76 (male=42)	79 (male=64)	127 (male=86)
**Gender ratio (m : f)**	2.16	1.24	4.27	2.01
**Age (years)**	53.3 ± 13.3	53.9 ± 12.1	52.8 ± 14.5	54.7 ± 13.4
**Age at onset (years)**	33.3 ± 15.8	36.1 ± 15.4	30.4 ± 15.9	28.6 ± 13.5
**Disease duration (years)**	20.7 ± 14.5	17.8 ± 13.9	23.5 ± 14.5	25.7 ± 15.3
**Typical episode duration (weeks)**			11.4 ± 8.5	11.1 ± 11.8
**Attack frequency (attacks in last week)**	12.7 ± 11.2	13.9 ± 11.2	11.5 ± 11.2	0
**Headache intensity (0-10)**	7.7 ± 2.2	8.1 ± 1.8	7.4 ± 2.2	8.9 ± 1.3
**Nocturnal attacks, n**	112 (72.2%)	59 (77.6%)	53 (67.0%)	112 (88.1%)
**Number of cranial autonomic symptoms**	4.9 ± 1.9	4.7 ± 1.9	5.1 ± 1.8	4.9 ± 2.0
**Restlessness during attacks, n**	151 (97.4%)	73 (96.1%)	78 (98.7%)	125 (98.4%)
**Use of acute medication, n**	154 (99.4%)	76 (100%)	78 (98.7%)	125 (98.4%)
**Number of different acute medications currently used**	4.1 ± 3.7	4.9 ± 3.3	3.3 ± 3.9	5.2 ± 3.6
**Number of different triptans currently used**	1.1 ± 1.3	1.1 ± 1.2	1.0 ± 1.3	1.7 ± 1.6
**Acute medication uses (in last week)**	6.9 ± 7.3	8.2 ± 8.0	5.7 ± 6.2	---
**Current use of preventive medication, n**	143 (91.0%)	69 (90.8%)	72 (91.1%)	114 (89.8%)*
**Number of different preventive medications currently used**	2.3 ± 2.3	2.8 ± 2.4	1.8 ± 2.0	2.6 ± 2.4*
**Current cigarette smoking, n**	43 (27.7%)	19 (25.0%)	24 (30.4%)	24 (18.9%)
**Current smokeless tobacco smoking, n**	8 (5.2%)	5 (6.6%)	3 (3.8%)	9 (7.1%)
**Current alcohol consumption, n**	66 (42.6%)	19 (25.0%)	47 (59.5%)	73 (57.5%)

The main reliability and validity analysis was based on 155 patients with ‘active’ CH (active episodic CH or chronic CH, 106 males; age 53.3 ± 13.3 years). These patients reported 12.7 ± 11.2 attacks/ week and 6.9 ± 7.3 acute medication uses/week in the baseline survey.

### Factor analysis

Data was suitable for factor analysis according to the KMO criterion (0.91) and Bartlett test (χ^2^(28) = 775.79, *p* < 0.001). Inspection of the scree plot and eigenvalues after principal axes factor analysis with oblimin rotation revealed one factor accounting for 62.69% of the variance. Factor loadings were meaningful for all items (0.57 to 0.88, Table 2).

**Table 2:** Item and factor analysis and test-retest correlation. Abbreviations:  CHIQ, Cluster Headache Impact Questionnaire; SD, standard deviation

	**Item statistics**				**Factor analysis**	**Test-retest reliability**		
	**Mean (SD)**	**Item difficulty**	**Corrected item-scale correlation (with item deleted)**	**Cronbach’s ** **a** ** (with item deleted)**	**Factor loading**	**Intraclass correlation**	**95% Confidence Interval**	
							**Lower Bound**	**Upper Bound**
**CHIQ1**	3.29 (1.26)	65.8	.80	.89	.87	.88	.78	.93
**CHIQ2**	3.43 (1.23)	68.6	.78	.89	.85	.90	.82	.95
**CHIQ3**	3.12 (1.39)	62.4	.75	.89	.83	.89	.80	.94
**CHIQ4**	3.23 (1.29)	64.6	.77	.89	.84	.75	.55	.86
**CHIQ5**	3.27 (1.35)	65.4	.70	.90	.78	.82	.64	.90
**CHIQ6**	2.79 (1.31)	55.8	.73	.90	.80	.83	.70	.91
**CHIQ7**	1.61 (1.48)	32.2	.49	.92	.57	.87	.75	.93
**CHIQ8**	3.01 (1.46)	60.2	.70	.90	.76	.82	.65	.91
**CHIQ score**						.93	.82	.96

### Item and scale analysis 

Results of the item analysis are shown in Table 2. Item difficulty was within the desired range (20–80%) and corrected item-scale correlations were good (with only item 7 slightly below 0.5) [18]. Internal consistency of the CHIQ was good with Cronbach’s α = 0.91. 

**Fig. 2:**
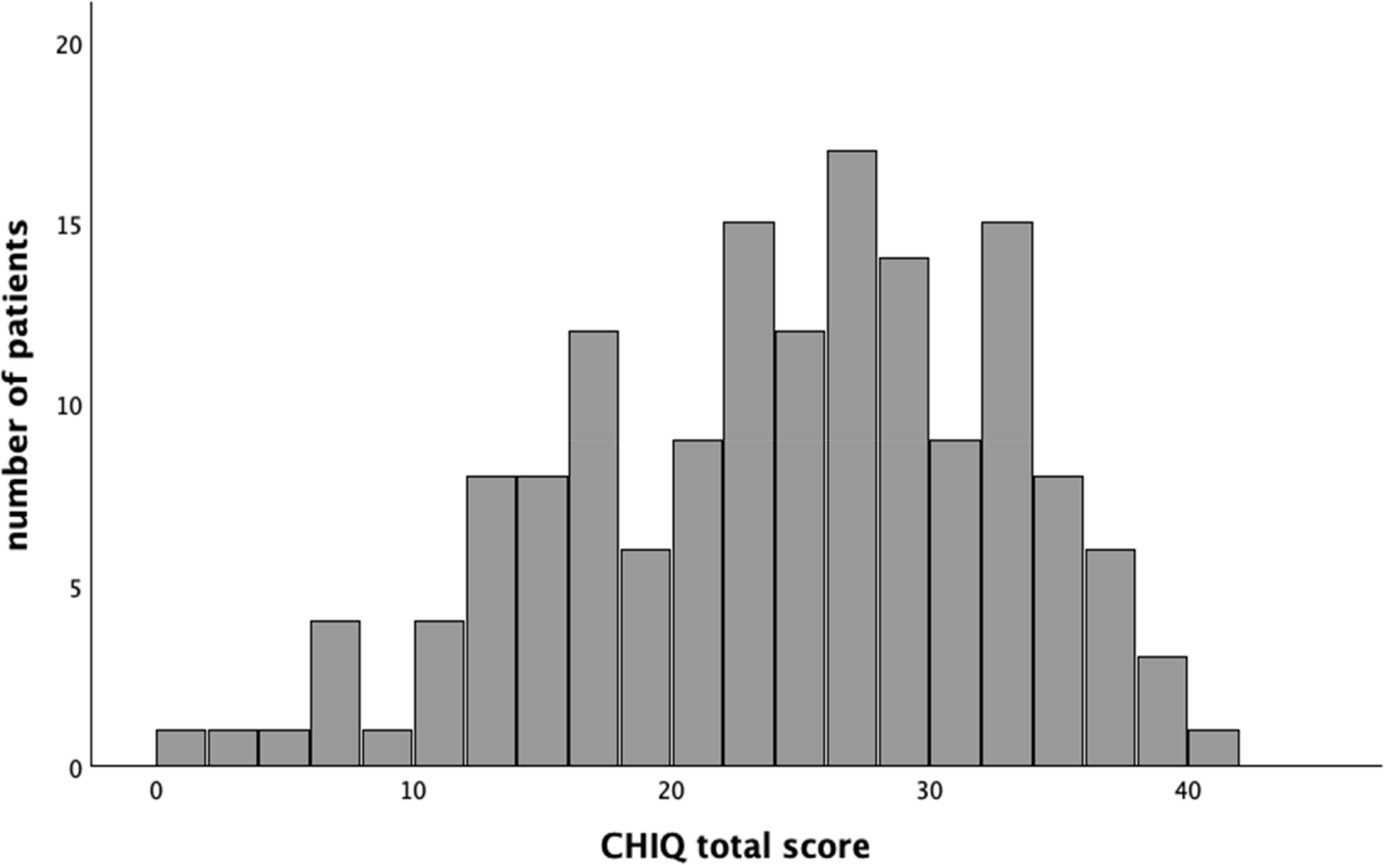
Histogram of CHIQ scores in ‘active CH patients’ (*n* = 155). Abbreviations: CH, cluster headache

The average CHIQ score was 23.7 ± 8.4 (possible range 0 - 40) in active patients. The histogram showed a slightly left-skewed distribution (Fig. 2) but no ceiling or bottom effects [22]. Accordingly, the Shapiro-Wilk-test revealed a significant deviation from normality (*p* < 0.05).

### Test‐retest reliability 

To assess test-retest reliability, we selected active patients with a maximum of two attacks/ week difference between test and retest. 44 patients fulfilled these criteria (15 eCH, 29 males, age 54.3 ± 11.9 years, test-retest interval 16.1 ± 3.2 days). Average CHIQ values were 23.9 ± 8.1 at test and 21.7 ± 9.1 at retest. Test-retest reliability was good (ICC=0.93). Test-retest correlations for single items (ICCs) were between 0.75 and 0.90 (see Table 2).

#### Convergent validity 

Convergent validity of the CHIQ was assessed by evaluating correlations with the HIT-6^TM^, the HADS and PSS-10 and are presented in Table 3. According to Cohen’s effect size graduation [19], significant positive correlations of large size were found with the HIT-6^TM^ (*ρ* = 0.72, *p* < 0.001), HADS depression and anxiety subscales (*ρ* = 0.53 and 0.61, both *p* < 0.001), and the Perceived Stress Scale (PSS-10, *ρ* = 0.61, *p* < 0.001). Correlations with attack frequency (*ρ* = 0.29, *p* < 0.001) and acute medication frequency (*ρ* = 0.21, *p* = 0.008) were significant and of small to medium size.

**Table 3:** Convergent validity. Spearman correlations in the active CH group (*n* = 155) are given. Abbreviations: CHIQ, Cluster Headache Impact Questionnaire; HADS, Hospital Anxiety and Depression Scale; HIT-6™, Headache Impact Test™; PSS, Perceived Stress Scale; SD, standard deviation

	**Mean ** **± SD**	**Correlation with CHIQ**
**Attack frequency (in last week)**	12.7 ± 11.2	0.29, p < 0.001
**Acute medication frequency (in last week)**	6.9 ± 7.3	0.21, p = 0.008
**HIT-6** **™**	63.3 ± 7.1	0.72, p < 0.001
**HADS anxiety**	8.6 ± 4.6	0.61, p < 0.001
**HADS depression**	7.7 ± 5.2	0.53, p < 0.001
**PSS-10 total score**	18.2 ± 8.4	0.61, p < 0.001
**PSS-10 helplessness**	11.8 ± 5.5	0.60, p < 0.001
**PSS-10 self-efficacy**	6.4 ± 3.3	0.57, p < 0.001

###  Group differences

**Fig. 3:**
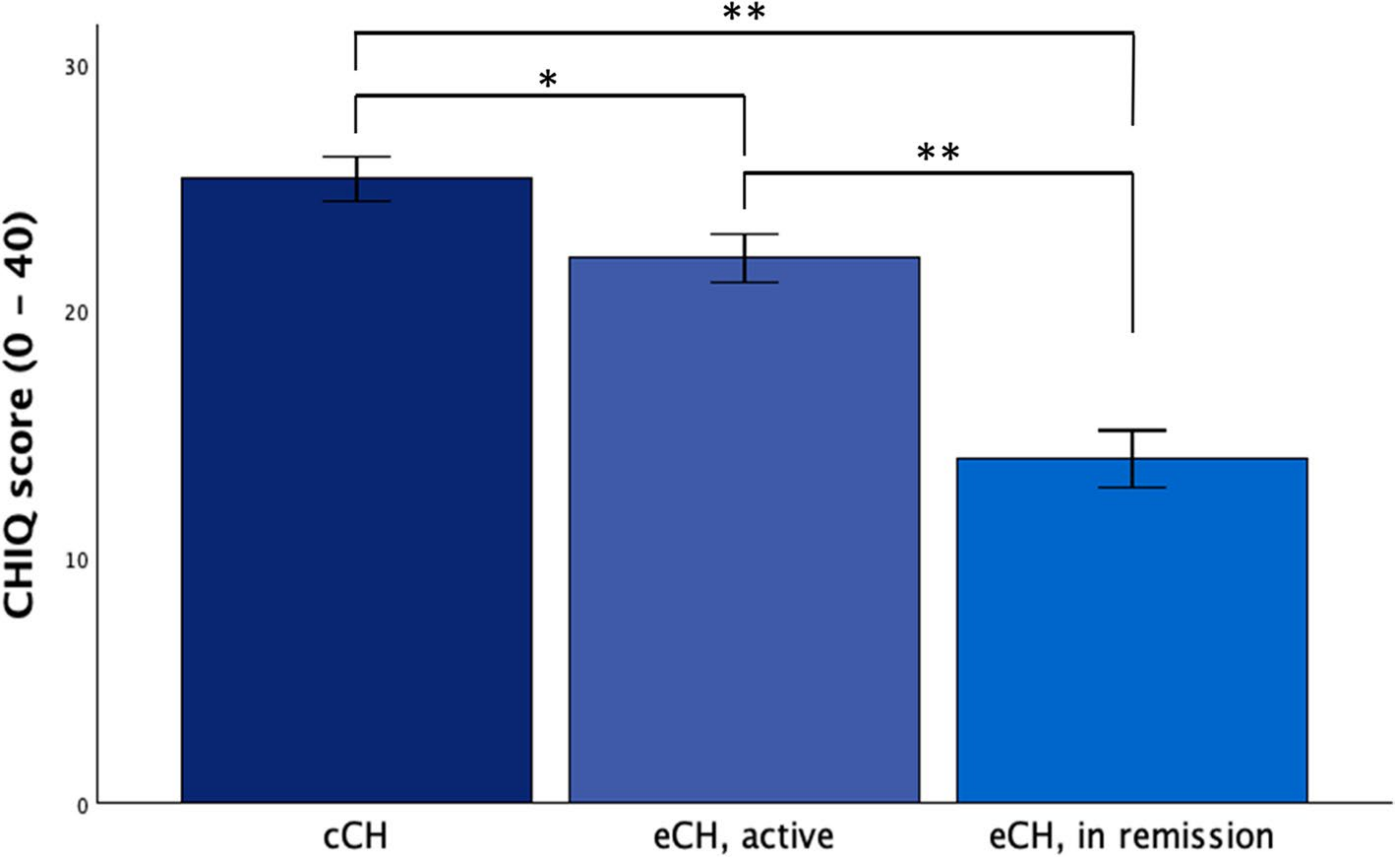
CHIQ score in CH patients. Chronic CH patients showed highest CH scores (25.4 ± 7.9, *n* = 76), followed by active episodic CH patients (22.2 ± 8.7, *n* = 79) and episodic CH patients in remission (14.1 ± 13.1, *n *= 127). The CHIQ scores are significantly different between the three groups (H[2] = 41.3, p < 0.001). *, *p* < 0.05; **, *p *< 0.001. Abbreviations: CH, cluster headache

Chronic CH patients showed the highest CHIQ scores (25.4 ± 7.9, *n* = 76) followed by active episodic CH patients (22.2 ± 8.7, *n* = 79) and episodic CH patients in remission (14.1 ± 13.1, *n* = 127, Fig. 3). Average CHIQ scores were significantly different between these patient groups (H[2] = 41.3, *p* < 0.001). Pairwise, Bonferroni-Holm corrected comparisons showed that all three groups were significantly different from each other (cCH vs. active eCH, *p* < 0.05; cCH vs. eCH in remission, *p* < 0.001; active eCH vs. eCH in remission, *p* < 0.001).

###  CHIQ grading

**Fig. 4:**
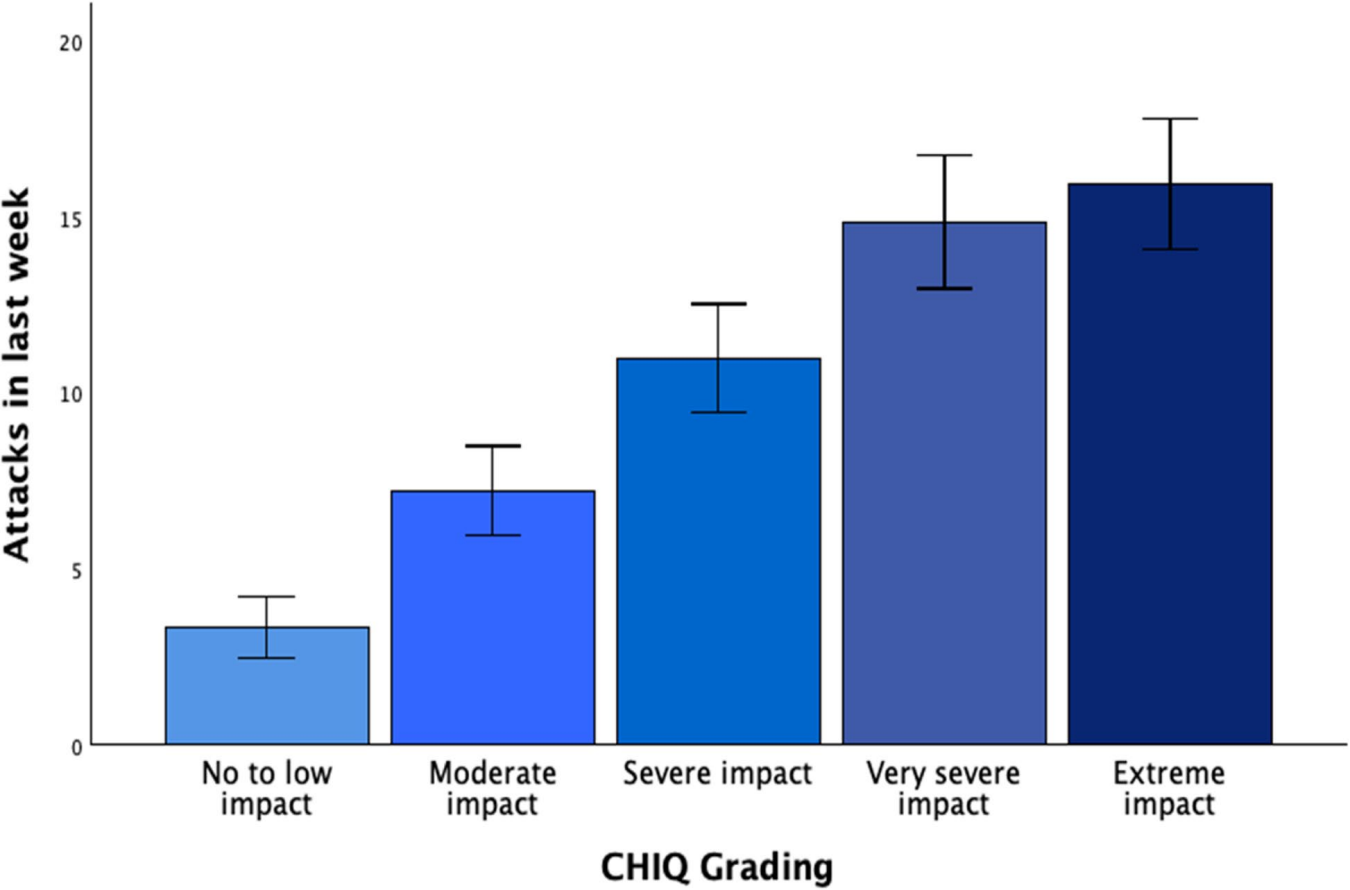
CHIQ grading. The CHIQ is graded into five categories from “no to low” to “extreme” impact according to the patients’ ratings of their subjective burden due to active CH. Patients rating their burden higher show higher attack frequency

To establish a labelled grading of the CHIQ, we considered both the requirement of a good discrimination in the upper half of the CHIQ scale where most active CH patients’ ratings are (see Fig. 2), and the necessity to assign a label that reflects the patients’ perception of impact. For the latter, we used the results of the single item question where active CH patients rated the impact of their CH during active episodes as not at all (*n* = 0), a little bit (*n* = 3), moderately (*n* = 18), quite a bit (*n* = 44) or extremely (*n* = 90). We propose a 5-step grading of the CHIQ, shown in Table 4 and Figure 4. Most active patients (*n* = 134, 86.5%) rated the impact as “quite a bit” or “extreme”, and all but 14 of these patients had a CHIQ rating ≥15, so we decided to divide CHIQ ratings between 15 and 40 into 3 groups with approximately equal numbers of patients, resulting in 15-23 points, 24-29 points and 30-40 points, which we labelled “severe”, “very severe” and “extreme”. On the other hand, there was no active patient rating CH impact as none and only 3 who rated the impact as “a little bit”, so we decided that only the lowest 5 points on the CHIQ scale should be graded as “no to low” impact. Between 5 and 14 points, we labelled the impact “moderate”. Table 4 also shows the distribution of single-item impact ratings among the different CHIQ grades. Attack frequency, HIT-6 scores, HADS and PSS scores increased monotonously with the grades, with significant differences corroborated by ANOVA (Table 4). Of the eCH patients in remission, 49 (38.6%) fell into grade 1, and 14, 27, 15 and 22 patients (11.0%, 21.3%, 11.8% and 17.3%) into grades 2, 3, 4 and 5, respectively.

**Table 4:** CHIQ grades. § Scale: none / a little bit / moderate / quite a bit / extreme; numbers of patients with the respective rating are given. Group comparisons were performed with Kruskal-Wallis ANOVA

Grade	1	2	3	4	5	Statistics
Grade label	No to low impact	Moderate impact	Severe impact	Very severe impact	Extreme impact	-
CHIQ result	0-4	5-14	15-23	24-29	30-40	-
N (in present sample)	3	23	44	43	42	-
Single item impact rating^§^	0 / 1 / 2 / 0 / 0	0 / 2 / 7 / 6 / 8	0 / 0 / 9 / 18 / 17	0 / 0 / 0 / 18 / 25	0 / 0 / 0 / 2 / 40	-
Attacks in last week	3.3 ± 1.5	7.2 ± 6.1	11.0 ± 10.2	14.9 ± 12.4	16.0 ± 12.0	H = 15.7, *p* = 0.003
Acute treatment uses in last week	2.7 ± 2.5	4.8 ± 4.6	6.7 ± 7.1	5.4 ± 7.2	10.0 ± 8.0	H = 16.5, *p* = 0.002
HIT-6 score	48.7 ± 2.9	56.8 ± 4.4	60.2 ± 6.0	65.1 ± 4.5	69.4 ± 5.5	H = 74.3, *P* < 0.001
HADS-anxiety	4.0 ± 2.0	5.6 ± 3.2	5.9 ± 3.3	9.6 ± 3.8	12.2 ± 4.2	H = 60.0, *P* < 0.001
HADS-depression	2.0 ± 3.5	4.7 ± 4.3	5.7 ± 4.0	8.1 ± 4.9	11.6 ± 4.5	H = 42.2, *P* < 0.001
PSS	11.0 ± 7.0	12.5 ± 6.6	13.7 ± 6.2	19.5 ± 7.5	25.3 ± 6.7	H = 56.0, *P* < 0.001

## Discussion

The present study demonstrates the reliability and validity of the English version of the CHIQ, with results comparable to those published for the original German version as well as the Italian version [7, 9].

The ‘active CH’ patient sample included in the present study (*n* = 155) was similar to that of the original German validation study (*n* = 196 [7]), with 49.0% vs. 43.4% chronic CH patients and 12.7 ± 11.2 vs. 15.2 ± 13.8 attacks/ week, respectively. These studies were also similar in that the majority of participants was recruited from a non-clinic based group (a community support group). In contrast, the Italian validation study included patients at their presentation to a tertiary headache center (*n* = 110, [9]), and exhibited a lower number of chronic CH patients (12.7%) and a median of 8 attacks/ week.

The present study confirmed the previous finding that the CHIQ consists of one factor, indicating that no meaningful subscales of the CHIQ could be identified (as intended) [7]. Internal consistency was good in all three studies (Cronbach’s α: present study: 0.91; German validation: 0.88; Italian validation: 0.89). Item statistics were generally good in all three studies, but revealed somewhat of an outsider position of item 7, which exhibited a lower average rating, and the lowest (although still adequate) values for item difficulty, corrected item-scale correlation and factor loading. Item 7 assesses self-injurious behavior, which might affect only a subgroup of patients, possibly explaining the somewhat weaker results. Nonetheless, self-injurious behavior is a feature of CH, so we decided to keep the item.

Test-retest reliability was good in the present (ICC = 0.93) and the German validation study (ICC = 0.91), while it was lower in the Italian validation study (ICC = 0.58). As the authors of the Italian study discuss, this might be due to patients starting treatment at the time of their baseline CHIQ assessment, which might have affected disability even in patients with similar number of attacks between test and retest. Test-retest reliability is notoriously difficult to assess in a rapidly changing disorder such as CH and further confirmation by additional studies would be desirable.

As expected, convergent validity was corroborated by high correlations between CHIQ scores and the generic headache disability questionnaire HIT-6^TM^ (present study: *ρ* = 0.72, German study: *ρ* = 0.58, no such questionnaire included in the Italian study). Correlations between the CHIQ and depression, anxiety and stress were also high (*ρ* = 0.53 to 0.61 in the present study, and *ρ* = 0.46 to 0.72 in the previous studies), showing that disability is tightly linked to psychological distress. Correlations with number of attacks and number of acute medication uses were significant, but of small to medium size in the present study (*ρ* = 0.21 to 0.29), similar to the Italian study (*ρ* = 0.15 to 0.19) while the previous German study showed somewhat larger correlations (*ρ* = 0.37 to 0.41). Together, the results illustrate that CH-related disability is a complex concept that goes beyond attack frequency and is tightly linked to measures of psychological distress.

Average CHIQ scores in active CH patients were remarkably similar in the three studies (23.7 ± 8.4 in the present study, 24.7 ± 6.8 in the German study, 24.8 ± 8.3 in the Italian sample). In the present study as well as in the German study, there was a small but significant difference in CHIQ scores between chronic CH patients and active episodic CH patients that was not found in the Italian study, maybe because of the low number of chronic CH patients in that study (*n* = 14). All three studies found highly significant differences between active CH patients and episodic CH patients in remission, which had average CHIQ scores of 14.1 ± 13.1 in the present study and 13.6 ± 11.9 in the German study. The Italian study found a higher CHIQ score in this group (median 21). We hypothesize that this could be due to patients presenting at the headache center shortly after the end of an episode, while CH patients recruited from a support group might have been in remission for a longer period. It would be an interesting follow-up analysis to assess if disability in CH patients in remission depends on the time since the end of the last episode. In any case, it is an important observation now corroborated by several studies, and also in non-specific disability questionnaires [20], that CH patients in remission still report significant disability due to CH. Disability in remission could reflect ongoing psychiatric comorbidity, as the CHIQ had significant positive convergent validity with the HADS depression and anxiety subscales. However, disability while in remission may also be due to other factors particular to CH, such as planning life activities while knowing relapse is probable.

Given the similarity of scoring between the German and English versions of the CHIQ, we here expand the preliminary German CHIQ grading to establish a final CHIQ grading with 5 grades. This final grading shows good distribution of the sample over the higher grades, allowing for discrimination, and labelling of the grades oriented at the overall ratings of the patients. We also showed that CH frequency, severity, psychological cofactors and disability assessed by the HIT-6™ highly correlated with the CHIQ grades.

### Other CH specific questionnaires

Recently, two other CH specific questionnaires have been developed, the 28-item Cluster Headache Quality of Life Scale (CHQ) [21] and the 36-item Cluster Headache Scales (CHS), that capture different psychosocial dimensions of CH [8]. These scales are elaborated tools comprising 4 and 8 subscales, respectively. They are well suited to research where CH-related quality of life and/or psychosocial dimensions are the main subject of study, but time constraints may limit their use in routine clinical care and in research where different questionnaires have to be assessed. For these applications, a brief questionnaire such as the CHIQ might be a good choice. More research is needed to compare the utility across these scales.

### Strengths and limitations

An important strength of our study is the large sample of 282 CH patients, of which 155 had active CH. Furthermore, we recruited from both clinic and non-clinic based populations. It is a limitation that for patients recruited via the community support group, CH diagnosis was self-reported, but we tried to compensate for that by assessing ICHD-3 criteria point-by-point within the headache questionnaire. Further, 71% of the patients stated having been diagnosed by a neurologist. As in our previous study, recruiting via a tertiary headache center and a support group might have led to overrepresentation of severely affected patients, also reflected by the high proportion of chronic CH patients (49%) compared to epidemiological data (~14% [22, 23]). Sensitivity to change (e.g., under treatment) has not been assessed yet and would be an important topic for a dedicated follow-up study. Finally, although elevated CHIQ scores suggest significant disability also in eCH patients in remission, this needs to be investigated in more detail, both regarding the reason for this on-going disability and the applicability of the CHIQ grading that was established based on active cluster headache patients.

## Conclusion

In conclusion, the present data show reliability and validity of the English language version of the CHIQ, and nicely matches data from previous CHIQ studies, demonstrating consistency of CHIQ properties over several samples and languages. Thus, the CHIQ is a decidedly short, valid and reliable assessment of CH related disability that can be used both in clinical practice and in research.

## Data Availability

No datasets were generated or analysed during the current study.

## Abbreviations

CH: Cluster headache
CHIQ: Cluster Headache Impact Questionnaire
CHQ: Cluster Headache Quality of Life Scale
CHS: Cluster Headache Scales
HADS: Hospital Anxiety and Depression Scale
HIT-6^TM^: Headache Impact Test
ICC: Intraclass correlation coefficient
ICHD-3: International Classification of Headache Disorders, 3^rd^ edition
KMO: Kaiser-Meyer-Olkin
MIDAS: Migraine Disability Assessment
PFA: Principal factor analysis
PSS-10: Perceived Stress Scale
SD: Standard deviation
SF12v2®: SF-12 Health Survey
